# Measuring Shape Parameters of Pearls in Batches Using Machine Vision: A Case Study

**DOI:** 10.3390/mi13040546

**Published:** 2022-03-30

**Authors:** Xinying Liu, Shoufeng Jin, Zixuan Yang, Grzegorz Królczyk, Zhixiong Li

**Affiliations:** 1 College of Mechanical and Electrical Engineering, Xi’an Polytechnic University, Xi’an 710060, China; 200211019@stu.xpu.edu.cn (X.L.); jdxyjsf@126.com (S.J.); 200211023@stu.xpu.edu.cn (Z.Y.); 2 Faculty of Mechanical Engineering, Opole University of Technology, 45-758 Opole, Poland; g.krolczyk@po.edu.pl; 3 Yonsei Frontier Lab, Yonsei University, Seoul 03722, Korea

**Keywords:** machine vision, intelligent agriculture, image segmentation, pit detection

## Abstract

To solve the problem of low precision of pearl shape parameters’ measurement caused by the mutual contact of batches of pearls and the error of shape sorting, a method of contacting pearls’ segmentation based on the pit detection was proposed. Multiple pearl images were obtained by backlit imaging, the quality of the pearl images was improved through appropriate preprocessing, and the contacted pearl area was extracted by calculating the area ratio of the connected domains. Then, the contour feature of the contact area was obtained by edge tracking to establish the mathematical model of the angles between the edge contour points. By judging the angle with a threshold of 60° as the candidate concave point, a concave point matching algorithm was introduced to get the true concave point, and the Euclidean distance was adopted as a metric function to achieve the segmentation of the tangent pearls. The pearl shape parameters’ model was established through the pearl contour image information, and the shape classification standard was constructed according to the national standard. Experimental results showed that the proposed method produced a better segmentation performance than the popular watershed algorithm and morphological algorithm. The segmentation accuracy was above 95%, the average loss rate was within 4%, and the sorting accuracy based on the shape information was 94%.

## 1. Introduction

Pearl shape is one of the important reference indicators of pearl quality. At present, the pearl shape parameters are mainly calculated by manually measuring the long and short axes of the pearls using a vernier caliper [1,2]. Although manual sorting has the advantages of high flexibility and comprehensive evaluation, the labor intensity is high, the efficiency is low, and the subjective factors make the sorting accuracy unstable [3]. In recent years, with the rapid development of machine vision technology, it has been feasible to use machine vision to replace manual measuring to eliminate the interference of subjective factors (such as fatigue and human emotions) and improve the measurement accuracy and efficiency for the pearl shape parameters [4]. In fact, accurate and rapid detection of pearl shapes has become an urgent need for pearl manufacturers. The machine vision technology has sufficient market prospects [5].

Existing literature shows that there are few research results in the pearl shape detection, particularly for batch pearls’ detection. Many detection methods are applied to a single pearl to measure the thickness of the pearl layer [6,7], pearl donor [8], internal structure [9,10], luster [11,12], color [13,14], etc. Although single pearl detection can analyze the surface and internal quality of the pearls from multiple angles [15], most of them are limited to laboratory environments. In addition, the market demands more for processing a batch of pearls at the same time; but batch pearl detection suffers from measurement accuracy due to mutual contact between pearls. Therefore, it is necessary to analyze and summarize the contact problems of batches of pearls.

For the issue of contacting target segmentation in different fields, researchers have proposed various segmentation algorithms, including the watershed algorithm [16,17], morphological algorithm [18,19], contour curve method [20,21,22], concave point method [23,24], and so on. These algorithms can achieve contact target segmentation for specific situations but, at the same time, they all have certain limitations. The watershed algorithm is a classic algorithm for segmenting overlapping images. It is fast, accurate, and effective, but it will be disturbed by noise to produce over-segmentation or under-segmentation, causing a large number of false edges, especially when multiple pearls are in contact. The morphological algorithm can easily destroy the original shape of the pearl, which is not conducive to the measurement of the pearl shape parameters in this paper. The contour curvature method and the curve fitting method have a large amount of calculation and low recognition rate and generally are only suitable for 2–3 pearls. The concave point method mainly uses the concave–convex characteristics of the target when it is in contact and divides the contact target according to the angle of the concave point; but the detection of the concave point is prone to errors. In comparison, the concave point method has more advantages, but its effect needs to be improved to increase the segmentation.

In summary, in view of the segmentation problem of contacting a large number of pearls in the shape detection, this paper proposes a new segmentation method based on the pit detection and target contact segmentation. Firstly, the backlit imaging was used to obtain a large number of pearl images and the area of pearl contact was extracted according to the area ratio of the connected domain mark. Secondly, a mathematical model of the angle between the contour points of the edge of the contact area was established to extract the concave point information, and the interference points were eliminated through the concave point matching. Then, the Euclidean distance was used as the distance measurement to complete the segmentation of tangent pearls. Lastly, the pearl contour and the position of the center of mass were calculated, and the pearl shape parameters’ model based on the image information was established to quantify the pearl shape. After the characterization was completed, the algorithm accuracy comparison and verification were performed.

We arranged this paper by the following. Section 1: For the pearl shape detection system, this paper selected the hardware required for robots and visual recognition and built an experimental platform. Section 2: The tangent pearls’ image was segmented based on concave point detection and concave point matching. Pearl shape parameters were measured by extracting the features of the contour edge. Section 3: Through the experimental platform built to conduct the experimental analysis of different numbers of pearl images contacted at different angles, we determined the pearl shape parameters and used the robot to complete the pearl classification according to the recognition results. Section 4: Conclusions.

## 2. Materials and Method

### 2.1. Experimental Platform

**Figure 1:**
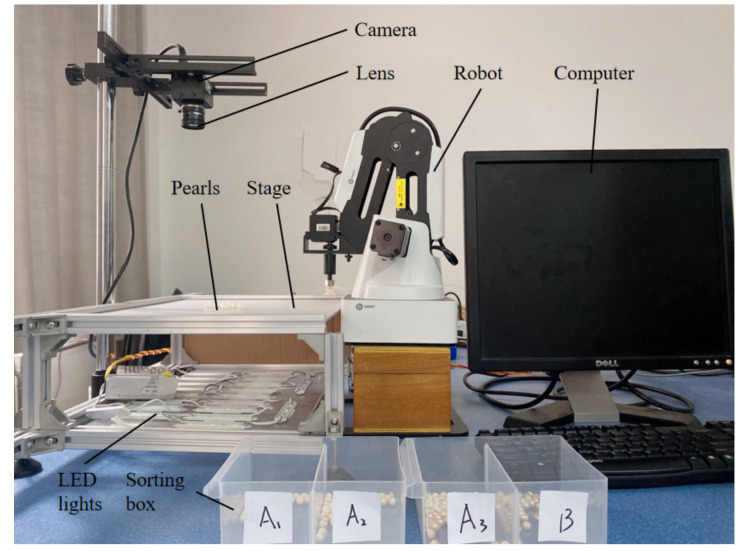
Pearl shape detection system platform.

A pearl shape detection system based on machine vision was developed, as shown in Figure 1, including a robot system, a vision detection system, a platform, a sorting box, and a computer. The visual inspection system consisted of a camera, lens, light source, computer, and the objective table. The camera was the Daheng Mercury camera MER-502-79U3M POL camera. The collected picture contained 20 pixels of 1 mm. The optical axis of the camera was perpendicular to the objective table. The lens was a computer series M2514-MP2 lens. The distance from the objective table was 250 mm. The pearls were randomly laid on the objective table. The light source adopted an LED array surface light source with an illuminance of 40,000 Lux. The camera, the pearl to be detected, and the light source acquired the pearl image with backlit imaging. The system software adopted Python3.7 and opencv3.4.2, and the computer performance was by CPU i7-11700k, 16-G memory, and a GTX 2080ti graphics card. A Dobot magician robot was adopted, and the end effector was a SMC-ZP3-T04BN-A5 vacuum suction cup.

**Figure 2:**
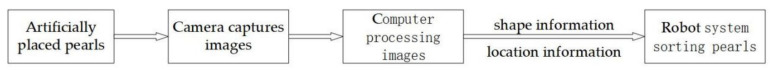
Operation flowchart.

The specific operation process for the pearl detection is shown in Figure 2.

### 2.2. Pearl Image Processing Method

Firstly, this work proposed a tangent pearls’ segmentation method based on an improved concave point detection for the boundary connectivity problem caused by batch pearls. Secondly, the tangent pearls’ area was extracted based on the connected domain mark. According to the principle of point matching, the true concave point pairs were screened out. Then, the tangent pearls were divided based on the Euclidean distance.

#### 2.2.1. Boundary Connectivity Problem

**Figure 3:**
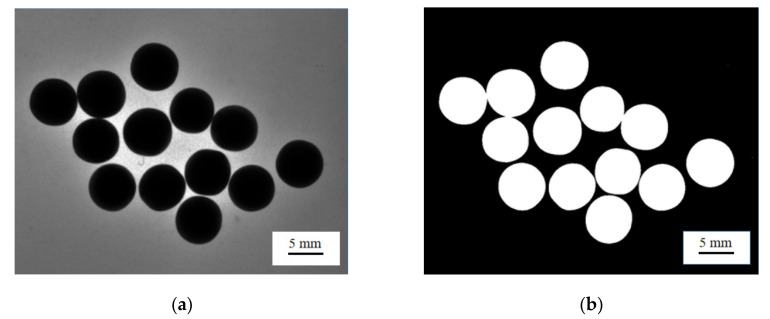
Pearl image preprocessing. (**a**) Original image of pearl; (**b**) pearl binary diagram.

There are texture and luster on the pearl surfaces [25]. In order to reduce the interference of these factors on the pearl shape detection, the backlit imaging was used to collect multiple pearl images, as shown in Figure 3a. In order to extract the pearls’ contour features, Gaussian filtering was performed after equalizing the histogram of Figure 3a, which preserved more edge information of the images while smoothing the image [26]. According to the gray distribution characteristics of the pearl images [27], the maximum between-class variance method [28] was used to segment the preprocessed pearl images and solved the problems of cavities and rough contour edges in the segmented pearl area. Linear structural elements were processed by the morphological algorithm [29]; the result is shown in Figure 3b.

It can be seen from Figure 3b that there were separate pearl outline areas in the divided pearl images, which were in contact with each other. To solve the segmentation problem of pearls in contact with each other, pit detection based on the three-point including angle and the pit matching method with adaptive characteristics were adopted.

#### 2.2.2. Extraction of Tangent Pearls’ Region

**Figure 4:**
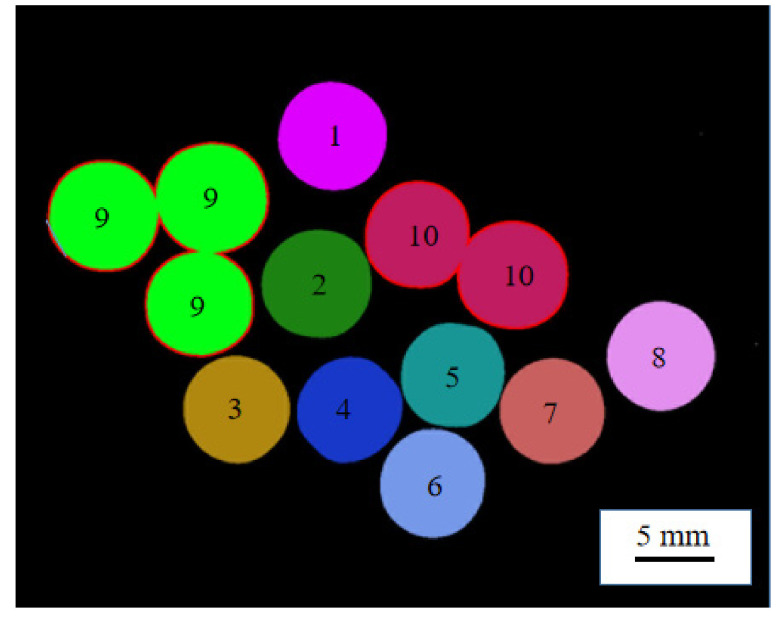
Connected domain labeling.

The connected domain labeling of the pearl images after binarization is shown in Figure 4. The pixel area of each connected domain was counted as $S_{1},S_{2},\cdots ,S_{n}$, as shown in Table 1. The area sequence was sorted and the smallest area was *S*_min_, as expressed by Equation (1).

(1) $$S_{\min}=\min\{S_{1},S_{2},\cdots ,S_{n}\}$$

where *n* is the number of connected domains.

Calculating the connected area of each connected domain is shown in Figure 4 and Table 1. The area of the smallest connected domain corresponded to a single, independent pearl. In order to obtain the pearl area in contact with each other, the ratio of the pixel area of each connected domain to the area of the smallest connected domain *S*_min_ was used to determine whether it was a contact area.

(2) $$K_{n}=\frac{\left\{S_{1},S_{2},S_{3}\cdots S_{n}\right\}}{S_{\min}}$$

where *K_n_* is the area ratio.

Since a large number of pearls were randomly laid out and there was no stacking state, it was necessary to select an area ratio as the threshold for judging whether the pearls were in contact. It can be seen from Table 1 that if S1–S8 were all less than 1.50, there were no tangent pearls in these eight areas; if S9 and S10 were both greater than 1.50, then there were pearls contacting each other in these two areas. Therefore, the area ratio 1.50 was selected as the threshold value. When *K_n_* > 1.50, the pearls were contacted; otherwise, they were independent. The edge tracking was used to extract the edge contour features of the pearls in contact with each other [30], as shown in contour nos. 9 and 10 in Figure 4.

#### 2.2.3. Image Segmentation

The concave point information is the point with the maximum value of curvature on the contour of the target edge in the image. The shape of a single, independent pearl is similar to the circle, and the surface curvature changes smoothly; therefore, it does not have the feature of concave points. Many pearls in contact with each other have large curvature mutations at the contact points and have obvious concave point characteristics. On the basis of extracting the edge contours of the pearls in contact with each other, this paper segmented the pearls in contact with each other based on an improved pit detection.

**Figure 5:**
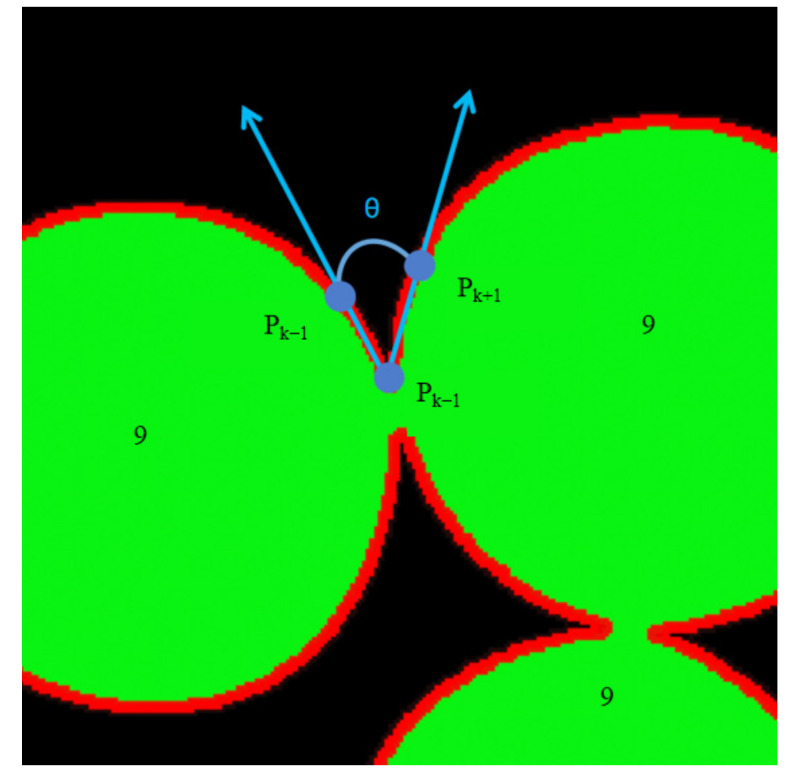
A partial enlargement of the contour of the tangent pearls’ edges.

The edge contour of the tangent pearls was firstly detected to obtain their edge contour sequence, as shown in Figure 5, from a partially enlarged view of the contour of the tangent pearls’ edges. A certain point was selected in the edge contour sequence as the current detection point, *p_k_*. The step size was set and then the coordinates of the point *p_k_*_+1_ were obtained. The step size was 2 pixels, and the coordinates of the three consecutive points were *p_k_*_−1_ (*x_k_*_−1_, *y_k_*_−1_), *p_k_* (*x_k_*, *y_k_*), and *p_k_*_+1_ (*x_k_*_+1_, *y_k_*_+1_).

The straight line *p_k_*_−1_*p_k_* intersected the straight line *p_k_p_k_*_+1_, and the obtained angle *θ* was the angle between the contour points of the pearl edge, as expressed by:

(3) $$S_{k-1}=|p_{k+1}p_{k}|=\sqrt{{\left(x_{k+1}-x_{k}\right)}^{2}+{\left(y_{k+1}-y_{k}\right)}^{2}}$$

(4) $$S_{k}=|p_{k+1}p_{k-1}|=\sqrt{{\left(x_{k+1}-x_{k-1}\right)}^{2}+{\left(y_{k+1}-y_{k-1}\right)}^{2}}$$

(5) $$S_{k+1}=|p_{k}p_{k-1}|=\sqrt{{\left(x_{k}-x_{k-1}\right)}^{2}+{\left(y_{k}-y_{k-1}\right)}^{2}}$$

According to the law of cosines, the angle *θ* is described as

(6) $$\theta =\frac{\arccos(S_{k+1}^{2}+S_{k-1}^{2}-S_{k}^{2})}{2S_{k+1}S_{k-1}}$$

**Figure 6:**
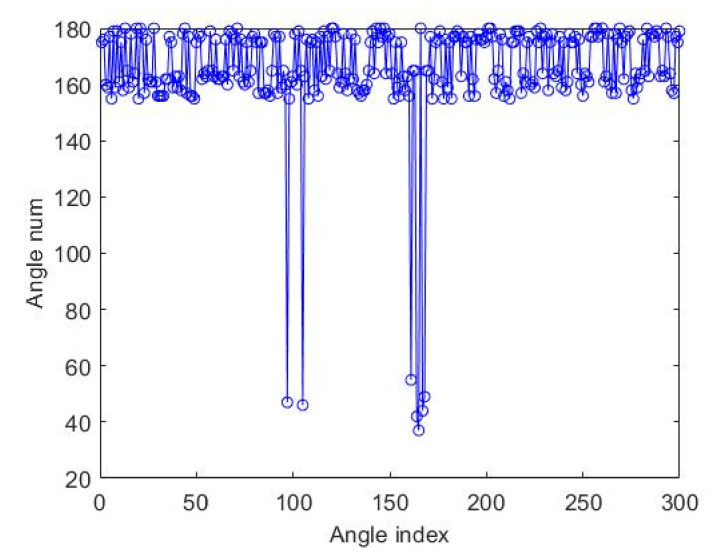
Angle distribution diagram.

Due to the texture of the pearl surface, the outline angle fluctuated and the actual concave point was relatively small. As shown in the angle distribution diagram shown in Figure 6 calculated by Equation (6), the general distribution of angles was within the range of 150°–80°, and the individual angles were within the range of 30°–60°, which can be used for initial pits’ determination.

**Figure 7:**
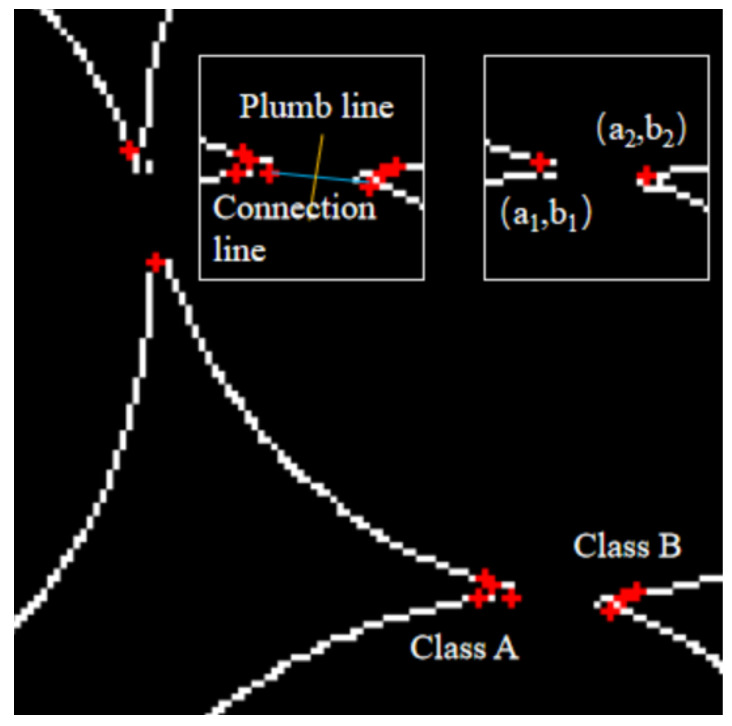
Preliminary pit detection.

The angle distribution in Figure 6 shows that there were interference points in the concave points determined by the included angle of the contour, and the preliminarily determined concave points were defined as the candidate concave points. In order to accurately determine the pits in the pearl contact areas, this paper developed a specific matching algorithm on the candidate pits; the steps were as follows:

(1) According to the distance between the candidate pits, the K-means clustering [31] was used to classify the candidate pits into classes A and B, as shown in Figure 7.
(2) We found a pair of candidate pits with the shortest distance between classes A and B.
(3) We connected the pair of candidate points and calculated the vertical line of the connecting contour.
(4) We set a minimum circle range with the length of the connecting line as the diameter and calculated the coordinates of the points of the mid-vertical line within this range.
(5) We found the gray value in the original image according to the acquired coordinates.
(6) If the gray value of the points was the gray of the pearl, then the pair of candidate pits were the real pits; if there were both the gray of the pearl and the gray of the background, it meant that the candidate pit contained only one true pit.
(7) If only one true pit was included, we increased the number of the candidate pits and returned to step (1).

Among them, the K-means clustering is a process of classifying and organizing data members that are similar in some respects.

The result of the concave point matching is shown in Figure 7; the pair of concave points (*a*_1_, *b*_1_) and (*a*_2_, *b*_2_) of the pearl contact point were accurately determined.

**Figure 8:**
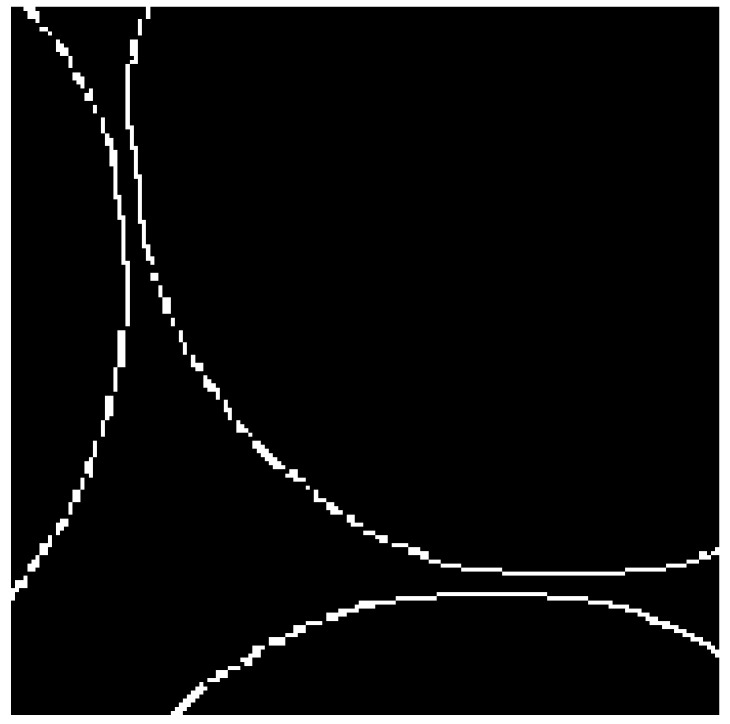
Tangent pearls’ segmentation.

Using the Euclidean distance as the distance metric function [32] $Dist[(a_{1},b_{1}),(a_{2},b_{2})]=\sqrt{{\left(a_{2}-a_{1}\right)}^{2}+{\left(b_{2}-b_{1}\right)}^{2}}$, the tangent pearls were segmented; the result is shown in Figure 8.

#### 2.2.4. Comparison of Different Image Segmentation Algorithms

In order to test the accuracy and stability of the proposed segmentation method, comparative experiments with different contact numbers and different segmentation methods were carried out, and the reliability evaluation parameters were used to analyze the results.

The segmentation accuracy rate *Y* and the loss rate of area *Z* were used to evaluate the segmentation accuracy of the tangent pearls.

(1) Segmentation accuracy

Assuming that the actual number of pearls in the pearl image is *L*, and the number of correctly segmented pearls is *C*, the expression for the correct rate of segmentation of the contact image is shown in Equation (7).

(7) $$Y=\frac{C}{L}$$

(2) The loss rate of area

Assuming that the area of pearl A is *S*_1_ and the area of pearl B is *S*_2_, then the sum of the areas in the non-contact state is (*S*_a_ = *S*_1_ + *S*_2_), and the total area in the contact state is *S*_b_.

The area ratio of pearl A in the non-contact state and the contact state is *K*_1_ and *K*_2_, and the expression is shown in Equation (8).

(8) $$K_{1}=\frac{S_{1}}{S_{a}},\;K_{2}=\frac{S_{2}}{S_{b}}$$

Thus, the expression of the loss rate of area *Z* is:

(9) $$Z=(1-\frac{K_{1}}{K_{2}})\times 100\%$$

The smaller the loss rate of area *Z* is, the higher the precision is of dividing the tangent pearls, and vice versa.

**Figure 9:**
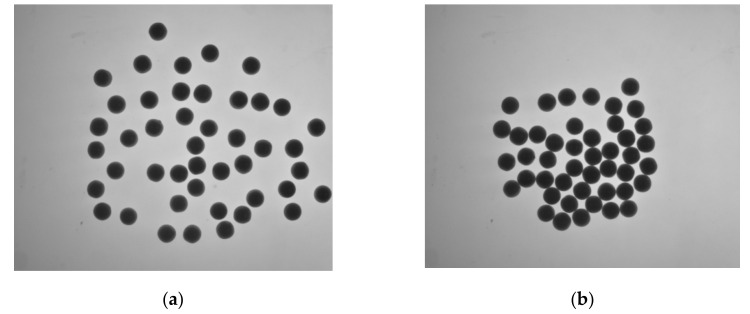
Pearls in two states. (**a**) Pearls in non-contact state; (**b**) contact state pearls.

As shown in Figure 9, taking multiple pearls as an example, Figure 9a is a non-contact state and Figure 9b is a contact state.

Based on the developed experimental platform in this paper, the watershed algorithm, the morphological algorithm, and the proposed method WERE compared with the experimental images. The differences of these three algorithms are discussed in Table 2.

**Figure 10:**
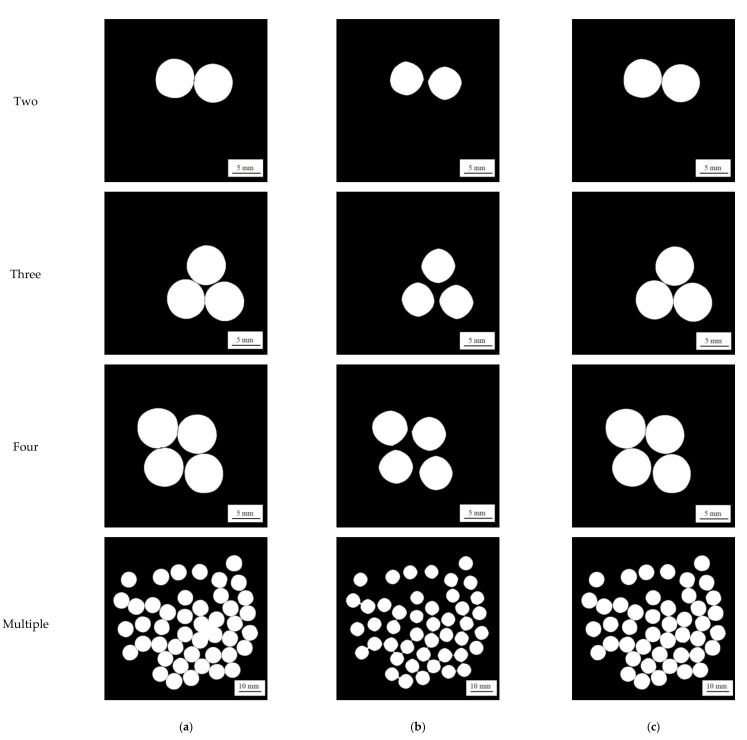
Different methods for different numbers of tangent pearls’ segmentation results. (**a**) Watershed algorithm; (**b**) morphological algorithm; (**c**) proposed algorithm.

The comparative results of the three algorithms are shown in Figure 10, and the comparison of segmentation results is shown in Table 3.

It can be seen from Table 3 that, compared with other methods, the proposed method was able to suppress under-segmentation and over-segmentation, and the segmentation effect was the best.

**Figure 11:**
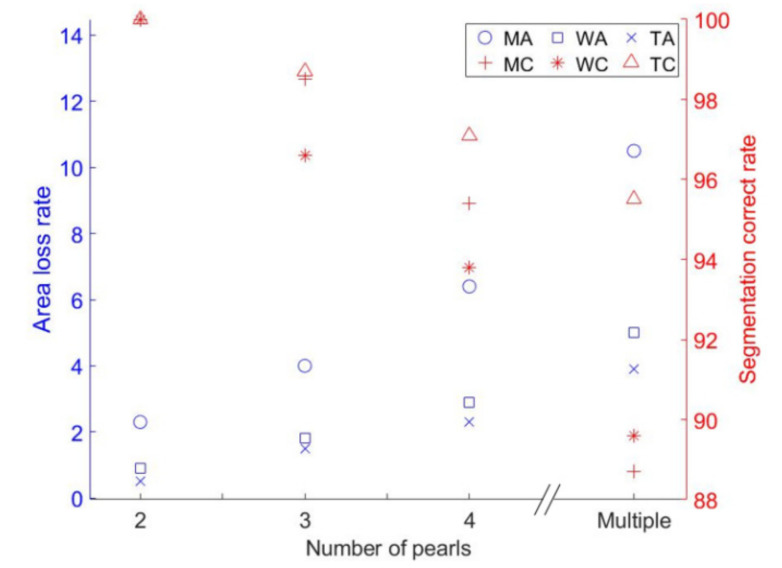
Comparison test statistical results of different segmentation methods.

Figure 11 shows the experimental segmentation performance of the three methods, where WA is the loss rate of area of the watershed algorithm, MA is the loss rate of area of the morphological algorithm, and TA is the loss rate of area of the proposed method, MC is the correct segmentation rate of the watershed algorithm segmentation, WC is the correct segmentation rate of the morphological algorithm, and TC is the correct segmentation rate of the proposed method. It can be seen from Figure 11 that the proposed method performed better than the other two methods in the comparison because its segmentation accuracy was less affected by the degree of contact and the average loss rate of area remained within 4%. As the number of tangent pearls increased, the segmentation accuracy of all three methods was reduced and the loss rate of the area was increased. When the number of the tangent pearls was two, all three methods achieved complete segmentation of the tangent pearls. When there were three tangent pearls, the correct segmentation rates of the proposed method, the morphological algorithm, and the watershed algorithm were 98.7%, 98.5%, and 96.6%, respectively, but the area loss rate of the morphological algorithm was higher than the others. When there were four tangent pearls, the correct segmentation rates of the proposed method, the morphological algorithm, and the watershed algorithm were, respectively, 97.1%, 95.4%, and 93.8%. When there were more pearls in contact, the segmentation accuracy of 95.5% for the proposed method was still the best and the loss rate of 3.9% was the least.

## 3. Experimental Results

### 3.1. Experimental Testing

In this paper, 200 pearls of different shapes were used to evaluate the shape parameters’ detection of the proposed method, including 39 round pearls, 52 round pearls, 58 near round pearls, and 51 oval pearls. According to the “National Standard of the People’s Republic of China GB/T18781-2008”, the size of a perfect circle, a circle, and nearly circular freshwater pearls is expressed by the minimum diameter. The sizes of other shapes of pearls are expressed by the maximum size multiplied by the minimum size. The pearl shape parameter is expressed by the diameter expressed as a percentage (%) in Equation (10).

(10) $$X=\frac{d_{\max}-d_{\min}}{\bar{d}}\times 100=\frac{\max(d_{i})-\min(d_{i})}{mean(d_{i})}\times 100$$

where *d*_max_ is the maximum diameter of the pearl, *d*_min_ is the minimum diameter of the pearl, $\bar{d}$ is the average of the maximum diameter and the minimum diameter, $d_{i}$ is any diameter, i is the number of pixels on the pearl outline; the smaller the diameter percentage is, the closer the pearl shape is to a circle, not vice versa. Taking a pearl with a diameter of 8 mm as an example and referring to the GB/T18781-2008 standard, the grading standards are shown in Table 4.

### 3.2. Experimental Results

There were 200 experimental pearls in the experiments. Firstly, the longest diameter and shortest diameter of each pearl were manually measured, the percentage differences between each pearl were calculated, and then all pearls were classified in advance as true values. Secondly, the diameter difference percentage of each pearl was obtained by the proposed method to classify the pearls. Lastly, the detection result of the proposed method was compared with the manual detection result and the performance evaluation index *F*1 was used for the multi-classification problem. The confusion matrix for binary classification problem is shown in Table 5.

The evaluation index *F*1 is calculated as follows.

(11) $$P=\frac{TP}{TP+FP}$$

(12) $$R=\frac{TP}{TP+FN}$$

(13) $$f1-score=\frac{1}{\frac{1}{2}(\frac{1}{P}+\frac{1}{R})}=\frac{2PR}{P+R}$$

where *P* is the precision rate, *R* is the recall rate, and the *f*1-*score* is the *F*1 value.

For a multi-classification problem, there are multiple confusion matrices. The *macro*-*F*1 method is used to calculate the *F*1 value.

(14) $$macro-P=\frac{1}{m}\sum_{i}P_{i}$$

(15) $$macro-R=\frac{1}{m}\sum_{i}R_{i}$$

(16) $$macro-F1=\frac{2\cdot macro-P\cdot macro-R}{macro-P+macro-R}$$

**Figure 12:**
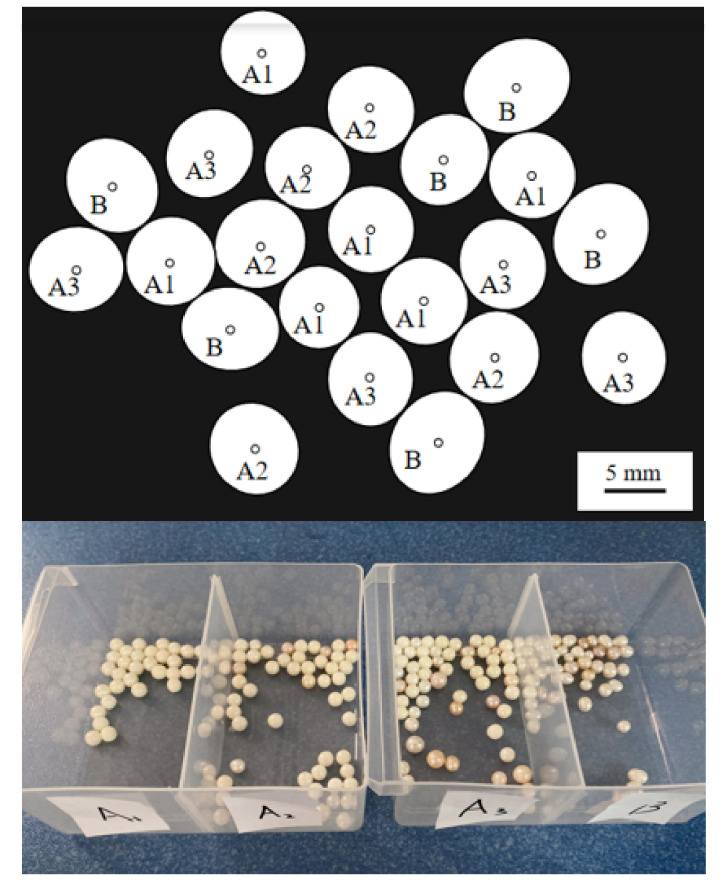
Robot sorting results.

The experimental results are shown in Table 6, Table 7, and Figure 12.

The experimental results showed that the proposed method produced a high detection accuracy. Among the four roundness pearls, the coincidence rate first decreased and then rose, and the lowest coincidence rate was reflected in the nearly circular shape. The trend of the coincidence rate was suspected to be caused by the differences in the pearl diameter because the diameter differences between the perfect circle and the ellipse were more obvious. The circle and the near circle were between the perfect circle and the ellipse, and the pearl diameter difference was small; therefore, there will be a certain error between the measurement results of the system software and the vernier caliper measurement results. In order to verify this conjecture, this work used some simulation pictures to contact different shapes of pearls at different angles, which simulated all possible contact situations of the pearls to detect the sensitivity of different pearl contact gestures.

**Figure 13:**
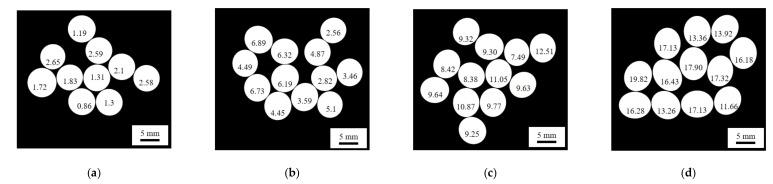
Recognition results of different shapes of pearls under different contact posture conditions. (**a**) Perfect circle; (**b**) Circle; (**c**) Near circle; (**d**) Ellipse.

Figure 13 shows the recognition results of different shapes of the pearls under different contact posture conditions. Using the proposed method to segment and detect simulated pearl images, when the pearls of different shapes were in contact at different angles, the detection coincidence rate of the perfect circle (a) and ellipse (d) was still high, respectively, i.e., 100% and 99.6%, and the detection coincidence rate of the circle (b) and near circle (c) was 97.8% and 97.1%. The coincidence rate between the artificial pearl shape detection and artificial comparison was consistent with the real pearl experiment results. It should be noted that since the behavior of the simulated pearls in this verification process was an ideal pearl, the coincidence rate of the simulation verification was higher than that of the real pearls.

## 4. Conclusions

To solve the problem of the multiple pearls in contact with each other in the pearl shape measurement, this paper proposed a new segmentation method based on pit detection. Compared with the watershed algorithm and the morphological algorithm, the proposed method was superior in the case of different numbers of tangent pearls; the segmentation accuracy of the proposed method was above 95% and the average loss rate of area was within 4%. Using simulated tangent pearls to investigate the sensitivity of different shapes of pearls with the proposed method under different contact angles, the coincidence rate of the round pearls was the highest, at 100%, and the coincidence rate of the nearly round pearls was the lowest, at 97.8%.

## Figures and Tables

**Table 1:** Connected domain area and area ratio.

Serial Number	1	2	3	4	5	6	7	8	9	10
Connected area (pixel)	14,193	14,382	14,064	13,249	13,434	13,068	13,211	13,751	41,467	25,796
*K_n_*	1.09	1.10	1.08	1.01	1.03	1.00	1.01	1.05	3.17	1.97

**Table 2:** The main differences between the three algorithms.

Algorithm	Watershed Algorithm	Morphological Algorithm	Algorithm of this Paper
Principles and ideas	The basic idea of the algorithm is to regard the image as a geodesic topological landform. The gray value of each pixel in the image represents the altitude of the point, and each local minimum and its affected area are called the catchment basin. The boundary of the catchment basin forms the watershed.	The basic idea of the algorithm is to measure and extract the corresponding shape in the image by using structural elements with a certain shape to achieve the purposes of image analysis and recognition.	The algorithm uses the concave points to describe the concave situation of the boundary, uses the boundary contour of the overlapping area to find the concave points, and finds the separation points from the concave points on the boundary to divide the overlapping area.
Advantage	The obtained boundaries are continuous with high accuracy and fast speed.	Good positioning effect, high segmentation accuracy, and good anti-noise performance. The basic morphological operations are erosion and dilation.	The calculation is simple; the features of the extracted points are uniform and reasonable; it is insensitive to image rotation, brightness changes, noise effects, and viewpoint changes.
Disadvantage	It has a good response to weak edges, noise in the image, and subtle grayscale changes on the surface of the object, which will cause over-segmentation.	After image processing, there are still a large number of short lines and isolated points that do not match the target. Due to the incomplete preprocessing work, a series of point-based opening (closing) operations are also required; so, the operation speed drops significantly.	It is sensitive to scale and has no geometric scale invariance. The extracted corners are pixel-level.

**Table 3:** Three algorithm segmentation results.

Algorithm	Watershed Algorithm (Figure 10a)	Morphological Algorithm (Figure 10b)	Algorithm of This Paper (Figure 10c)
Segmentation	It can segment obviously tangent pearls. When multiple pearls are seriously tangent, the binary image cannot extract the background area features at the location where the pearls are tangent and then cannot extract the segmentation endpoints, resulting in under-segmentation.	It is possible to achieve better segmentation of tangent pearls; but, since corrosion and expansion are not reversible operations, it can be clearly seen that the pearls’ shapes have changed significantly.	The selection of candidate concave points in the tangent pearl image is “adaptive”. When it is determined that all the current candidate concave points are near the real concave points, the follow-up candidate points will continue to be searched until a new candidate point appears, which avoids the difficulty of using it. Determined algorithm parameters can remove the interference of pseudo-pits.

**Table 4:** Pearl shape level.

Pearl Shape	Perfect Circle A1	Circle A2	Near Circle A3	Ellipse B
Diameter range (mm)	8 ≤ $d_{i}$ ≤ 8.24	8 ≤ $d_{i}$ ≤ 8.67	8 ≤ $d_{i}$ ≤ 9.02	7.2 ≤ $d_{i}$ ≤ 8.8
Percentage difference in diameter (%)	≤3.0	≤8.0	≤12.0	≤20.0

**Table 5:** Confusion matrix for binary classification problem.

Real Result	Forecast Result	
	Positive Example	Counter Example
Positive example	*TP* (real example)	*FN* (false counter example)
Counter example	*FP* (false positive)	*TN* (true counter example)

**Table 6:** Evaluation index.

Accuracy (P)	Recall Rate ^®^	*F*1 Value
0.95	0.94	0.95

**Table 7:** Pearl grade classification results.

Pearl Grade	Machine Inspection (Pieces)	Manual Detection (Pieces)	Consistency Rate (%)
Perfect circle A1	39	39	100.0
Circle A2	54	52	96.3
Near circle A3	55	58	94.8
Ellipse B	52	51	98.1

## Data Availability

All data used in this work can be requested from the corresponding author.

## References

[1] Ho J.W.Y., Shih S.C. (2021). Pearl Classification: The Gia 7 Pearl Value Factors. Gems Gemol..

[2] Xuan Q., Fang B., Liu Y., Wang J., Zhang J., Zheng Y., Bao G. (2018). Automatic Pearl Classification Machine Based on a Multistream Convolutional Neural Network. IEEE Trans. Ind. Electron..

[3] Bai F., Fan M., Yang H., Dong L. (2021). Image Segmentation Method for Coal Particle Size Distribution Analysis. Particuology.

[4] Yaghoobi H., Mansouri H., Farsangi M.A.E., Nezamabadi-Pour H. (2019). Determining the Fragmented Rock Size Distribution Using Textural Feature Extraction of Images. Powder Technol..

[5] Cao Y.L., Zheng H.W., Yang J.X., He Y.F. (2010). Automatic Shape Grading of Pearl Using Machine Vision Based Measurement. Key Eng. Mater..

[6] Ju M.J., Lee S.J., Min E.J., Kim Y., Kim H.Y., Lee B.H. (2010). Evaluating and Identifying Pearls and Their Nuclei by Using Optical Coherence Tomography. Opt. Express.

[7] Ju M.J., Lee S.J., Kim Y., Shin J.G., Kim H.Y., Lim Y., Yasuno Y., Lee B.H. (2011). Multimodal analysis of pearls and pearl treatments by using optical coherence tomography and fluorescence spectroscopy. Opt. Express.

[8] Agatonovic-Kustrin S., Morton D.W. (2012). The Use of UV-Visible Reflectance Spectroscopy as an Objective Tool to Evaluate Pearl Quality. Mar. Drugs.

[9] Toyota T., Nakauchi S. (2013). Optical Measurement of Interference Color of Pearls and Its Relation to Subjective Quality. Opt. Rev..

[10] Nagata N., Dobashi T., Manabe Y. (1997). Modeling and Visualization for a Pearl-Quality Evaluation Simulator. IEEE Trans. Vis. Comput. Graph..

[11] Satitkune S., Monarumit N., Boonmee C., Phlayrahan A., Promdee K., Won-In K. (2016). Combination of FTIR and SEM for Identifying Freshwater-Cultured Pearls from Different Quality. Opt. Spectrosc..

[12] Monarumit N., Noirawee N., Phlayrahan A., Promdee K., Won-In K., Satitkune S. (2015). Identification of High-Luster and Lusterless Freshwater-Cultured Pearls by X-Ray Absorption Spectroscopy. J. Appl. Spectrosc..

[13] Xuan Q., Chen Z., Liu Y., Huang H., Bao G., Zhang D. (2019). Multiview Generative Adversarial Network and Its Application in Pearl Classification. IEEE Trans. Ind. Electron..

[14] Ozaki R., Kikumoto K., Takagaki M., Kadowaki K., Odawara K. (2021). Structural colors of pearls. Sci. Rep..

[15] Loesdau M. (2016). Towards a Computer Vision Based Quality Assessment of Tahitian Pearls: Automatic Nacre Thickness Measurement and Color Classification, Computer Science. Ph.D. Thesis.

[16] Liang Y., Fu J. (2019). Watershed Algorithm for Medical Image Segmentation Based on Morphology and Total Variation Model. Int. J. Pattern Recognit. Artif. Intell..

[17] Zhang C., Shen X., Cheng H., Qian Q. (2019). Brain Tumor Segmentation Based on Hybrid Clustering and Morphological Operations. Int. J. Biomed. Imaging.

[18] Lei T., Jia X., Zhang Y., He L., Meng H., Nandi A.K. (2018). Significantly Fast and Robust Fuzzy C-Means Clustering Algorithm Based on Morphological Reconstruction and Membership Filtering. IEEE Trans. Fuzzy Syst..

[19] Lei T., Jia X., Liu T., Liu S., Meng H., Nandi A.K. (2019). Adaptive Morphological Reconstruction for Seeded Image Segmentation. IEEE Trans. Image Processing.

[20] Yu Y., Cao H., Wang Z., Li Y., Li K., Xie S. (2019). Texture-and-Shape Based Active Contour Model for Insulator Segmentation. IEEE Access.

[21] Zhou Z., Dai M., Wang T., Zhao R. (2019). Prior Distribution-Based Statistical Active Contour Model. Multimed. Tools Appl..

[22] Dan Z., Philip C.C., He Y., Tieshan L. (2019). Multi-Scale Adaptive Level Set Segmentation Method Based on Saliency. IEEE Access.

[23] Wang D., Fu Y., Yang G., Yang X., Liang D., Zhou C., Zhang D. (2019). Combined Use of FCN and Harris Corner Detection for Counting Wheat Ears in Field Conditions. IEEE Access.

[24] Luo L., Liu W., Lu Q., Wang J., Wen W., Yan D., Tang Y. (2021). Grape Berry Detection and Size Measurement Based on Edge Image Processing and Geometric Morphology. Machines.

[25] Wang Z., Adzigbli L., Zheng Z., Yang C., Deng Y. (2020). How Cultured Pearls Acquire Their Colour. Aquac. Res..

[26] McGuinness B., Duke M., Au C.K., Lim S.H. (2021). Measuring Radiata Pine Seedling Morphological Features Using a Machine Vision System. Comput. Electron. Agric..

[27] Heriawan M.N., Koike K. (2015). Coal quality related to microfractures identified by CT image analysis. Int. J. Coal Geol..

[28] Sung H.-J., Park M.-K., Choi J.W. (2020). Automatic Grader for Flatfishes Using Machine Vision. Int. J. Control. Autom. Syst..

[29] Liu L., Lim S., Shen X., Yebra M. (2019). A multiscale morphological algorithm for improvements to canopy height models. Comput. Geosci..

[30] Lapico A., Sankupellay M., Cianciullo L., Myers T., Konovalov D.A., Jerry D.R., Zenger K.R. (2019). Using Image Processing to Automatically Measure Pearl Oyster Size for Selective Breeding. Digit. Image Comput. Tech. Appl. DICTA.

[31] Sinaga K.P., Yang M.-S. (2020). Unsupervised K-Means Clustering Algorithm. IEEE Access.

[32] Cardarilli G.C., Di Nunzio L., Fazzolari R., Nannarelli A., Re M., Spanò S. (2020). N-Dimensional Approximation of Euclidean Distance. IEEE Trans. Circuits Syst. II Express Briefs.

